# Adrenergic Metabolic and Hemodynamic Effects of Octopamine in the Liver

**DOI:** 10.3390/ijms141121858

**Published:** 2013-11-05

**Authors:** Andrea Luiza de Oliveira, Mariana Nascimento de Paula, Jurandir Fernando Comar, Vanessa Rodrigues Vilela, Rosane Marina Peralta, Adelar Bracht

**Affiliations:** Department of Biochemistry, University of Maringá, Avenida Colombo 5790, Maringá 87020900, Brazil; E-Mails: andreabiomed@gmail.com (A.L.O.); mnpaulafarma@gmail.com (M.N.P.); jfcomar@uem.br (J.F.C.); vanexinha00@hotmail.com (V.R.V.); rmperalta@uem.br (R.M.P.)

**Keywords:** liver, octopamine, glycogenolysis, gluconeogenesis, oxygen uptake

## Abstract

The fruit extracts of *Citrus aurantium* (bitter orange) are traditionally used as weight-loss products and as appetite suppressants. A component of these extracts is octopamine, which is an adrenergic agent. Weight-loss and adrenergic actions are always related to metabolic changes and this work was designed to investigate a possible action of octopamine on liver metabolism. The isolated perfused rat liver was used to measure catabolic and anabolic pathways and hemodynamics. Octopamine increased glycogenolysis, glycolysis, oxygen uptake, gluconeogenesis and the portal perfusion pressure. Octopamine also accelerated the oxidation of exogenous fatty acids (octanoate and oleate), as revealed by the increase in ^14^CO_2_ production derived from ^14^C labeled precursors. The changes in glycogenolysis, oxygen uptake and perfusion pressure were almost completely abolished by α_1_-adrenergic antagonists. The same changes were partly sensitive to the β-adrenergic antagonist propranolol. It can be concluded that octopamine accelerates both catabolic and anabolic processes in the liver via adrenergic stimulation. Acceleration of oxygen uptake under substrate-free perfusion conditions also means acceleration of the oxidation of endogenous fatty acids, which are derived from lipolysis. All these effects are compatible with an overall stimulating effect of octopamine on metabolism, which is compatible with its reported weight-loss effects in experimental animals.

## 1. Introduction

Octopamine is produced from tyramine and has been identified as a naturally occurring biogenic amine in invertebrates and vertebrates [1]. This amine, originally found as a natural component extracted from the posterior salivary glands of *Octopus vulgaris* [2,3], is an important neurotransmitter in insects [4,5]. In mammalians, octopamine is present in the sympathetically innervated organs and the brain [1]. Its concentrations in these mammalian tissues, however, are very low and it may be considered a trace amine. In invertebrates, however, octopamine is present at relatively high concentrations in neuronal as well as in non-neuronal tissues and it modulates a great number of physiological processes to the point that it can be considered the invertebrate counterpart of epinephrine [6,7].

Besides its role as a biogenic amine, octopamine has been identified as an important active component in herbal products of *Citrus aurantium* [8] and other citrus species [9,10]. The fruit of *C. aurantium*, commonly known as bitter orange, is sometimes used as a food, but it is more widely ingested as a medicinal or dietary supplement. Extracts of *C. aurantium* have been claimed to promote weight loss. The active ingredients of *C. aurantium* include various alkaloids with adrenergic activity, including synephrine and octopamine [11,12]. Structurally, these active components in *C. aurantium* are closely related to endogenous neurotransmitters and ephedrine. Synephrine is similar in structure to epinephrine and octopamine is similar to norepinephrine [13].

The similarity to well-known adrenergic compounds, and the fact of being an active ingredient of *C. aurantium*, used for weight loss purposes, led to studies about the lipolytic activity of octopamine in adipocytes. It was found that rat brown adipocytes respond to octopamine by increasing oxygen consumption and lipolysis [14]. Recently it was shown that octopamine and synephrine activate lipolysis both in rat and human adipocytes [15]. These amines, however, are much less efficient in human than in rat adipocytes. Evidence has been presented that octopamine is a β_3_-antagonist in mammalian fat cells [14].

Although a fair number of investigations have been already published about the effects of *C. aurantium*, synephrine and octopamine on mammalian fat cells, much less attention has been given to their possible effects on the liver cells. The ingestion of *C. aurantium* preparations for weight loss purposes is obviously directed toward the fat cells, but one cannot avoid metabolic actions on other tissues, especially on the liver, which is the metabolic organ par excellence and the first that receives orally ingested compounds via the portal vein. A recent first approach to the effects of the *C. aurantium* amines in the rat liver has shown that commercial extracts are able to affect several hepatic metabolic variables. The effects include stimulations of glycogen catabolism (glycogenolysis) and oxygen uptake and inhibition of gluconeogenesis [16]. Hemodynamic effects were observed as well, more precisely, a pronounced increase in the portal perfusion pressure. The effects on glycogenolysis, oxygen uptake and hemodynamics are sensitive to α_1_- and β_2_-adrenergic antagonists, but are insensitive to the β_3_-antagonist SR59230A [16]. Also, most of the effects of the *C. aurantium* extract were reproduced by *p*-synephrine. The main exception was gluconeogenesis inhibition, which was not found with *p*-synephrine and is thus exerted by other compounds in the *C. aurantium* extract. On the other hand, the action of octopamine on liver metabolism is unkown, as no experiments on the subject have so far been reported. As an adrenergic agonist, however, it is likely that octopamine affects several metabolic routes, as has indeed been found for oxygen uptake in fat cells, which is stimulated by the compound [15]. It has also been reported that in insect muscles, octopamine activates glycolysis by increasing the levels of fructose 2,6-bisphosphate, a potent activator of the glycolytic key enzyme phosphofructokinase [17]. To characterize the metabolic effects of octopamine in the liver was, thus, the main purpose of the present work. This was accomplished by measuring several basic metabolic routes in the isolated perfused rat liver, a preparation which preserves the microcirculation and also allows the measurement of hemodynamic parameters such as the portal perfusion pressure [18]. The results of this investigation should therefore lead to an increased understanding of the effects of octopamine in mammalian organisms.

## 2. Results and Discussion

### 2.1. Effects of Octopamine on Glycogen Catabolism, Oxygen Uptake and Hemodynamics

**Figure 1:**
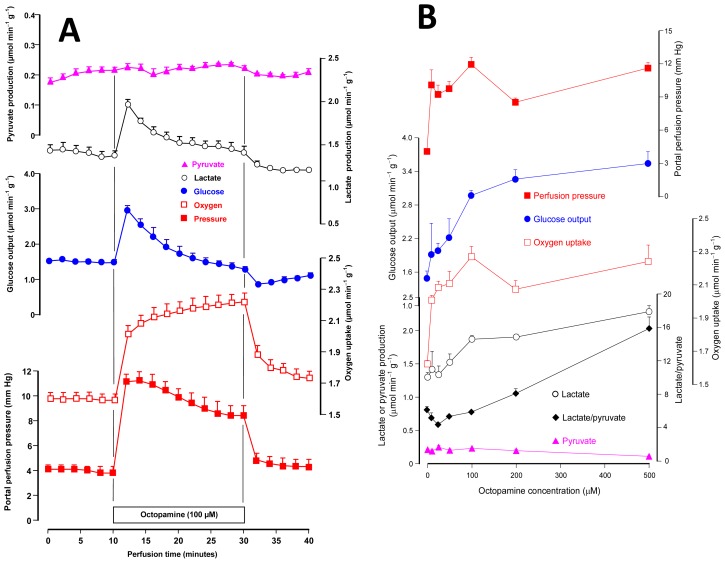
Effects of octopamine on the perfusion pressure, glycogen catabolism and oxygen uptake in the perfused liver of fed rats. Livers were perfused with substrate-free perfusion fluid as described in the experimental section. **Panel A** illustrates the time courses of the changes caused by 100 μM octopamine. And **Panel B** shows the concentration dependences of the changes caused by octopamine between 10 and 500 μM. Datum points are means ± mean standard errors of 3–4 liver perfusion experiments.

The first experiments were planned to test the effects of octopamine on carbohydrate catabolism and oxygen uptake in the liver of fed rats. In these experiments it was also investigated if octopamine is active in hemodynamics, because this kind of action is typical for compounds that act as adrenergic agents [19]. Livers from fed rats, where perfusion is conducted with substrate-free medium, survive at the expense of the oxidation of endogenous fatty acids (major route) and glycolysis from endogenous glycogen (minor route). Under these conditions, the organ releases glucose, lactate and pyruvate as a result of glycogen catabolism [20]. Figure 1A illustrates the time course of the responses of the perfused rat liver to the infusion of octopamine at the concentration of 100 μM. It also illustrates a typical experimental protocol, which was used for all other octopamine concentrations. After a pre-perfusion period of 10 min, 100 μM octopamine was infused during 20 min. This was followed by additional 10 min of octopamine-free perfusion. Six parameters were measured: glucose release, lactate and pyruvate productions, oxygen consumption and the portal perfusion pressure. All these parameters were stable before the initiation of octopamine infusion. An immediate response occurred in most parameters, however, when the infusion was initiated. The perfusion pressure increased to a maximal value of 11.1 ± 0.6 mm Hg at 14 min perfusion time. It declined slowly thereafter, but was still considerably above the basal levels at the end of the octopamine infusion (30 min perfusion time). Oxygen uptake was gradually stimulated, tending to a new steady state that was 0.61 ± 0.02 μmol·min^−1^·g^−1^ above the basal rates (*p* = 0.001, *n* = 3). Glucose output was rapidly increased with a peak increment of 1.49 ± 0.02 μmol·min^−1^·g^−1^ (*p* = 0.0002, *n* = 3) at 2 min after initiation of the infusion. It declined progressively and, at the end of the infusion, it was equal to the basal levels. Lactate production presented the same profile, with a peak increment of 43% at 2 min after starting octopamine infusion. Pyruvate production was not significantly affected by 100 μM octopamine. The effects were reversible; that is, cessation of the octopamine infusion resulted in termination of the effects.

In order to investigate the concentration dependence of the effects, experiments such as those illustrated by Figure 1A were repeated with several octopamine concentrations in the range between 10 and 500 μM. The results are presented in Figure 1B and represent the maximal values of each parameter during the infusion of octopamine as a function of the portal octopamine infusion. For oxygen uptake this always means the value found at the end of the octopamine infusion (30 min perfusion time) because the oxygen uptake increment was always stable. For the other parameters the decline after the peak value deaccelerated with the concentration and was minimal at the concentration of 500 μM. The first curve on the top of Figure 1B shows the concentration dependence of the portal perfusion pressure. There is some fluctuation with the various concentrations, but the increment was essentially the same for all concentrations in the range from 10 to 500 μM. The portal pressure is, thus, highly sensitive to octopamine. Oxygen uptake is also highly sensitive to octopamine as there was only a small difference between the increment caused by 10 μM and all other concentrations. Glucose output and lactate production stimulations, however, were a saturable function of the octopamine concentration in the range between 10 and 500 μM. The concentrations for half-maximal effects were equal to 70.7 and 87.0 μM, respectively, as obtained by numerical interpolation. The increase in lactate production, combined with the small diminution in pyruvate production, resulted in increased lactate to pyruvate ratios at high octopamine concentrations (200–500 μM). The lactate to pyruvate ratio reflects the cytosolic NADH/NAD^+^ ratio [21], meaning thus an increased availability of reducing equivalents in the cytosol.

It seems appropriate at this point to compare the action of octopamine on the hepatic glycogenolysis in the rat with its action on the analogous process in invertebrates. Evaluation of glycogen phosphorylase activation by octopamine in the fat body of the cockroach *Blaberus discoidalis* revealed approximately 50% of maximal activation at a concentration of 1 μM [22]. This means that in *Blaberus discoidalis* octopamine is approximately 70-fold more potent as a glycogenolytic agent than in the rat liver as can be judged from the concentration for half-maximal stimulation of glucose output mentioned in the preceding paragraph. It should be remarked, however, that oxygen uptake stimulation and vasoconstriction in the liver are more sensitive to octopamine than glycogenolysis, because for the former maximal or nearly maximal effects were found at the concentration of 10 μM. The more accentuated glycogenolytic activity of octopamine in insects contrasts with the more pronounced activity of epinephrine in the rat liver. In the latter, for example, maximal or nearly maximal activation of glycogenolysis is already achieved with 10 μM epinephrine (or norepinepehrine) [23–25], whereas 100 μM norepinephrine is required for full activation of glycogen phosphorylase in *Blaberus discoidalis* [22]. These and other observations are consistent with the generally accepted idea that in invertebrates octopamine exerts the analogous role of epinephrine or norepinephrine in vertebrates [6].

Glycolysis stimulation by octopamine, which in the present work is revealed by the increased rates of lactate output, seems also to occur in invertebrates. In *Locusta migratoria* flight muscle, for example, octopamine increases, in a concentration dependent manner, the levels of fructose 2,6-biphosphate [17], the key activator of glycolysis.

### 2.2. Effects of Octopamine on Gluconeogenesis and the Associated Oxygen Uptake

**Figure 2:**
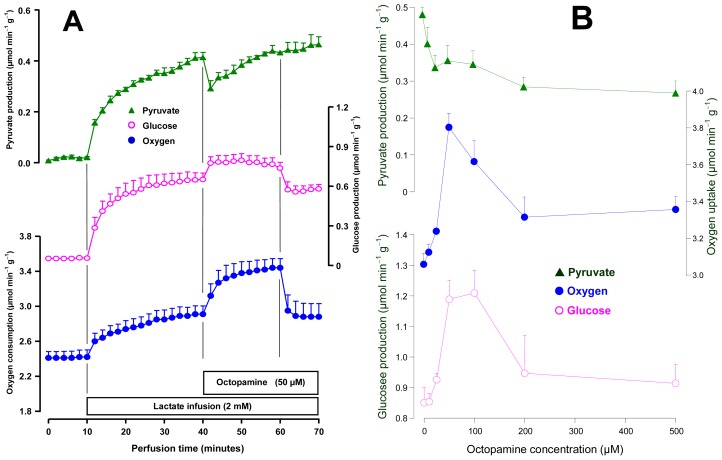
Effects of octopamine on lactate gluconeogenesis and associated parameters in the perfused liver of fasted rats. Livers were perfused as described in the experimental section. Lactate and octopamine were infused as indicated. **Panel A** illustrates the time courses of the changes caused by 500 μM octopamine. And **Panel B** shows the concentration dependences of the changes caused by octopamine between 10 and 500 μM. Datum points are means ± mean standard errors of 3–4 liver perfusion experiments.

Livers from 18 h fasted rats were perfused for evaluating the effects of octopamine on gluconeogenesis and the associated oxygen uptake increment. For this purpose 2 mM lactate was used as a gluconeogenic substrate. Figure 2A shows the mean results obtained with 50 μM octopamine. After stabilization of oxygen consumption, lactate was infused during 30 min followed by an additional period of 20 min in which 2 mM lactate plus 50 μM octopamine were infused. The following parameters were measured: oxygen uptake and the productions of glucose and pyruvate. The infusion of 2 mM lactate caused immediate increases in all parameters. At 30 min perfusion time they had already reached new steady states. The infusion of octopamine immediately raised oxygen uptake, from 2.90 ± 0.17 to 3.61 ± 0.07 μmol·min^−1^·g^−1^ (*p* = 0.019, *n* = 3). This stimulation remained so during the whole infusion time of octopamine. Glucose production was stimulated by approximately 40% during the whole infusion period. The pyruvate production was initially diminished (−24%) but the diminution was followed by a progressive recovery. The effects were reversible, that is, there was a clear tendency of returning to the basal values after cessation of octopamine infusion.

The effects of octopamine on gluconeogenesis were also examined for its concentration dependence in the range between 10 and 500 μM. The mean results are shown in Figure 2B. Stimulation of oxygen uptake was maximal at the concentration of 50 μM and declined thereafter. Glucose production stimulation also presented a maximum, but in the range from 50 to 100 μM. It should be noted that stimulation of both oxygen uptake and glucose production seemed to have stabilized in the concentration range between 200 and 500 μM. The inhibition of pyruvate production shown in Figure 2B as a function of the octopamine concentration actually represents the initial decline that followed immediately the onset of the octopamine infusion (see Figure 1A). The extent of this transient decline was concentration-dependent, as can be seen in Figure 2B. The difference in the effects of octopamine on glucose output in the fed state (saturation curve; Figure 1B) and fasted state (maximum followed by decrease; Figure 2B) can be explained by the different sources of the carbohydrate under the two conditions: glycogen degradation in the fed state and gluconeogenesis in the fasted state. The similar difference in the responses of oxygen uptake, on the other hand, can be explained in the same way if one assumes that the increment caused by octopamine on oxygen uptake in the fasted state is at least partly coupled to the oxidation of the reducing equivalents resulting from lactate oxidation. In fact, at least partial correlations between oxygen uptake increment and gluconeogenesis from lactate have been reported [26]. Experiments with the *C. aurantium* extract revealed inhibition of gluconeogenesis [16]. No inhibition was found with octopamine even at concentrations as high as 500 μM, although this was not the concentration for which maximal stimulation of gluconeogenesis was found (Figure 2B). In the experiments that were done with the *C. aurantium* extract the octopamine concentrations were certainly much lower. It can thus be concluded that octopamine is not responsible for the gluconeogenesis inhibition reported for the *C. aurantium* extract.

### 2.3. Effects of Octopamine on Fatty Acid Metabolism

**Figure 3:**
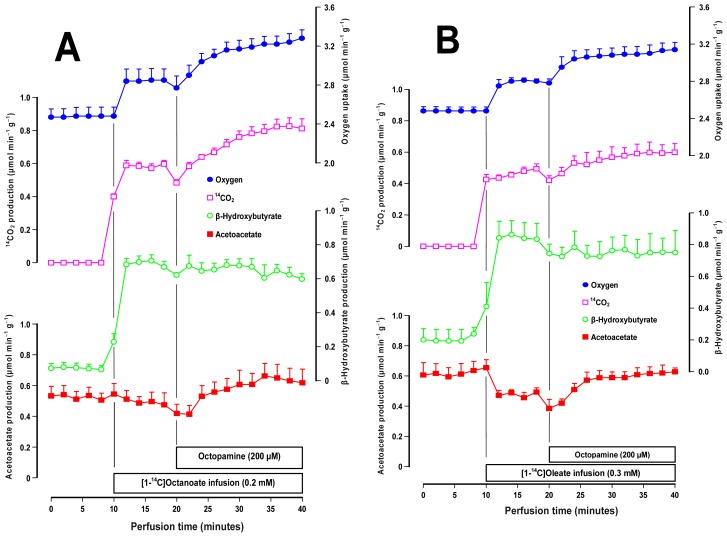
Effects of octopamine on medium- and long-chain fatty acid metabolism. Livers of fasted rats were perfused as described in the experimental section. **Panel A** illustrates the time courses of the changes caused by 200 μM octopamine during 0.2 mM [1-^14^C]octanoate infusion and **Panel B** the time courses of the changes caused by 200 μM octopamine during 0.3 mM [1-^14^C]oleate infusion. [1-^14^C]oleate was dissolved into the Krebs/Henseleit-bicarbonate buffer containing 0.15 mM fatty acid-free bovine-serum albumin. Datum points are means ± mean standard errors of 3–4 liver perfusion experiments.

Oxygen uptake in substrate-free perfused livers is driven essentially by the oxidation of endogenous fatty acids [27]. Since octopamine stimulates oxygen uptake under these conditions, it is of interest to know whether it also stimulates oxidation of exogenous fatty acids. In the present work this question was investigated using the ^14^C-labeled forms of a medium- and a long-chain fatty acid, namely [1-^14^C]octanoate and [1-^14^C]oleate. Figure 3A shows the time courses of the experiments that were done with 0.2 mM [1-^14^C]octanoate and 200 μM octopamine. Four parameters were measured: oxygen uptake, ^14^C-production, β-hydroxybutyrate and acetoacetate production. Since livers from fasted rats were used, there was a significant basal production of acetoacetate and β-hydroxybutyrate. The introduction of [1-^14^C]octanoate increased oxygen uptake and β-hydroxybutyrate production. It also caused the appearance of ^14^CO_2_ and a small diminishing tendency of acetoacetate production. The octopamine infusion further increased oxygen uptake (plus 0.40 ± 0.05 μmol·min^−1^·g^−1^; *p* = 0.013). It also increased the ^14^CO_2_ production (plus 0.36 ± 0.09 μmol·min^−1^·g^−1^; *p* = 0.032) and the acetoacetate production (plus 0.16 ± 0.02 μmol·min^−1^·g^−1^; *p* = 0.011), but did not affect the β-hydroxybutyrate production. The β-hydroxybutyrate/acetoacetate ratio, which is an indicator for the mitochondrial NADH/NAD^+^ ratio [21] was decreased by octopamine from 1.47 ± 0.17 to 1.02 ± 0.09 (*p* = 0.034).

Figure 3A shows the results obtained with labeled oleate. In general terms the response of the liver to the [1-^14^C]oleate infusion was similar to that found when [1-^14^C]octanoate was infused except that the former caused a well defined decrease in acetoacetate production. The response to the subsequent octopamine infusion was also similar but the changes were less pronounced. Oxygen uptake was increased by 0.31 ± 0.07 μmol·min^−1^·g^−1^ (*p* = 0.026) and the ^14^CO_2_ production by 0.14 ± 0.04 μmol·min^−1^·g^−1^ (*p* = 0.018). The acetoacetate production increment (0.13 ± 0.05 μmol·min^−1^·g^−1^) lacked statistical significance at the 5% level (*p* = 0.075). The decrease in the β-hydroxybutyrate/acetoacetate ratio (from 1.53 ± 0.06 to 1.21 ± 0.11), however, was statistically significant (*p* = 0.033).

The results in both panels of Figure 3 show that octopamine is also able to increase the oxidation of exogenous fatty acids in the liver. The fact that the mitochondrial NADH/NAD^+^ ratio, as indicated by the β-hydroxybutyrate/acetoacetate ratio [21], was decreased indicates that the mitochondria became more efficient in oxidizing NADH in the presence of octopamine. This observation suggests that octopamine stimulates the mitochondrial respiration not solely by increasing the availability of reducing equivalents but also by increasing the intrinsic activity of the respiratory chain.

### 2.4. Influence of Adrenergic Antagonists on the Effects of Octopamine

**Figure 4:**
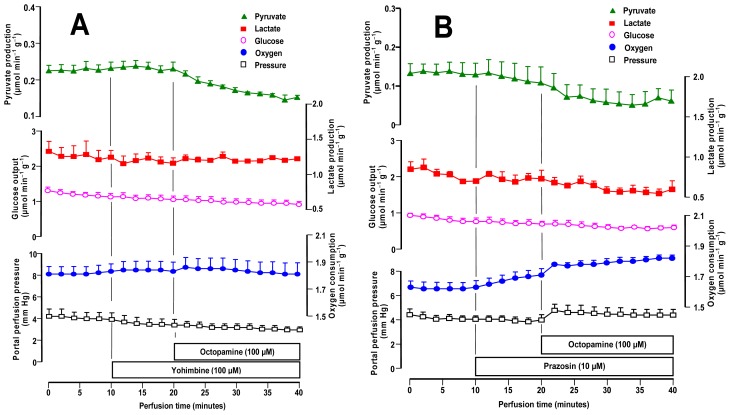
Influence of yohimbine (**Panel A**) and prazosin (**Panel B**) on the metabolic and hemodynamic effects of octopamine in livers from fed rats. Livers were perfused as described in Materials and Methods. The experimental protocols and the portal concentrations of yohimbine, prazosin and octopamine are indicated by the bars just above the time scale. Datum points are means ± mean standard errors of 3 liver perfusion experiments.

For verifying if the metabolic and hemodynamic effects of octopamine in the liver are mediated by adrenergic receptors, we performed experiments using several antagonists: (a) yohimbine (α_1_ and α_2_-antagonist), (b) prazosin (mainly α_1_-antagonist) [28–30] and (c) propranolol (non-specific β-antagonist) [31,32]. Livers from fed rats were used in all experiments. The action of 100 μM yohimbine in the rat liver is shown in Figure 4A. Yohimbine alone was without any effect on the variables measured in the present experiments. With respect to the effects of octopamine they were practically abolished by yohimbine, including those on oxygen uptake. Only pyruvate production inhibition was still detectable.

The results obtained with the α_1_-antagonist prazosin (10 μM) plus octopamine (100 μM), are shown in Figure 4B. The effects of prazosin alone, which was infused before octopamine, were not important except perhaps for a small stimulation of lactate production. When octopamine was infused in the presence of 10 μM prazosin no changes in glucose release or lactate production were found. Prazosin, thus, completely abolished the actions of octopamine on these variables. Pyruvate production was still inhibited to a small extent by octopamine and oxygen uptake and perfusion pressure still suffered small increments. However, these effects were much smaller than those ones found in the absence of prazosin (compare Figures 1A and 4B).

**Figure 5:**
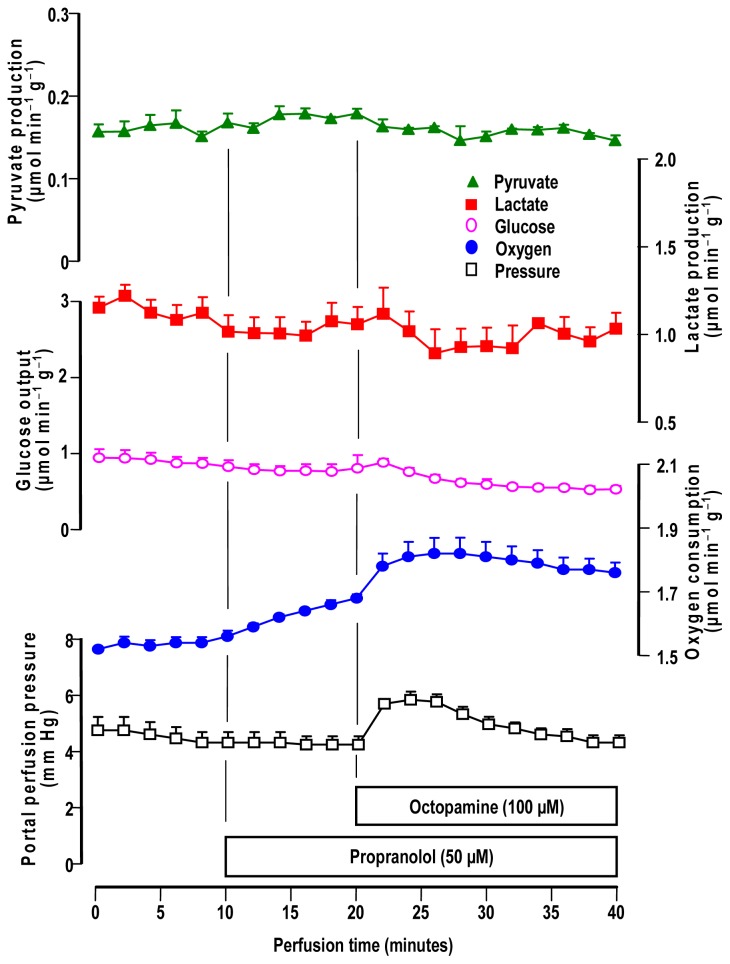
Influence of propranolol on the metabolic and hemodynamic effects of octopamine in livers from fed rats. Livers were perfused as described in materials and methods. The experimental protocols and the portal concentrations of propranolol and octopamine are indicated by the bars just above the time scale. Datum points are means ± mean standard errors of 5 liver perfusion experiments.

The influence of 50 μM propranolol, a β_1_- and β_2_-adrenergic antagonist, is illustrated by Figure 5. Propranolol alone caused an increase in oxygen uptake, but the other parameters were not affected. The introduction of 100 μM octopamine produced a further stable increase in oxygen uptake. The perfusion pressure was also increased by octopamine but to a lesser extent when compared to the increase observed in the absence of propranolol (compare Figures 1 and 5). Glucose release and the lactate and pyruvate productions, in contrast, were no longer affected by octopamine in the presence of propranolol.

The effects of the adrenergic antagonists on the action of octopamine are similar to those found for the action of the same antagonists on the effects of the *C. aurantium* extract [16] and they indicate an action mechanism mediated by adrenergic receptors for the majority of the effects. The high sensitivity of the effects of octopamine to 10 μM prazosin is highly indicative of the predominant participation of α_1_-adrenergic receptors [28]. The same can be said about the sensitivity to 100 μM yohimbine because at this concentration the latter compound, usually regarded as a α_2_-antagonist, also binds to α_1_-adrenergic receptors [28]. The participation of β-adrenergic receptors seems to be less important, as can be judged, in principle at least, from the fact that propranolol was less effective in inhibiting the action of octopamine than the α_1_-adrenergic antagonists. The effects via α_1_- and β-adrenergic receptors are not occurring independently, however. If α_1_- and β-adrenergic signalling occur independently, the summation of the metabolic responses in the presence of prazosin (or yohimbine) and propranolol should correspond to the metabolic responses in the absence of antagonists. This is evidently not the case, because yohimbine and prazosin completely eliminated the effects of octopamine on glucose output and oxygen uptake. Cross-talk events between α_1_- and β-adrenergic signalling are thus likely [33]. The mechanism or mechanisms underlying these interrelationships, however, cannot be deduced from the present data and need to be elucidated by further experimental work.

Octopamine also binds to 5-hydroxytryptamine receptors (5-HT receptors) and possibly to the newly discovered trace amine associated receptors (TAARs) [34–36]. The 5-HT receptors, can also be antagonized by the same antagonists used in the present work [37]. The question arises, thus, whether the 5-hydroxytryptamine receptors could be mediating the hepatic effects of octopamine. Concerning the metabolic effects (e.g., glycogenolysis stimulation), this possibility is rather remote, as short-term metabolic effects mediated by 5-HT receptors have not yet been reported, at least not for the liver. Vasoconstriction, however, is among the effects mediated by the 5-hydroxytryptamine and trace amine associated receptors. The vasoconstrictive action of octopamine observed in the present work could thus be partly mediated by one of these receptors. This is in line with the conclusion drawn from a recent study with the rat aorta [36]. In this preparation vasoconstriction by low octopamine concentrations is due predominantly to a direct or indirect action at α_1_-adrenergic receptors, but at higher concentrations other receptors might also be involved [36].

## 3. Experimental Section

### 3.1. Materials and Animals

The liver perfusion apparatus was built in the workshops of the University of Maringá. Octopamine, adrenergic antagonists, enzymes and coenzymes used in the enzymatic assays were purchased from Sigma Chemical Co. (St. Louis, MO, USA). All other chemicals were from the best available grade.

Male Wistar rats weighing 200–280 g were used in all experiments. Animals were fed *ad libitum* with a standard laboratory diet (Nuvilab^®^, Colombo, Brazil) and maintained on a regulated light-dark cycle. In accordance with the requirements of the experimental protocols, fed rats as well as 18 h fasted rats were used. For the surgical procedure, the rats were anesthetized by intraperitoneal injection of sodium pentobarbital (50 mg/kg). The criterion of anesthesia was the lack of body or limb movement in response to a standardized tail clamping stimulus. All experiments were done in accordance with internationally accepted ethical guidelines for animal experimentation.

### 3.2. Liver Perfusion

Hemoglobin-free, non-recirculating perfusion was performed [20,38]. After cannulation of the portal and cava veins, the liver was positioned in a plexiglass chamber. The constant perfusate flow was provided by a peristaltic pump (Minipuls 3, Gilson, France) and adjusted between 30 and 33 mL/min, depending on the liver weight. The perfusion fluid was Krebs/Henseleit-bicarbonate buffer (pH 7.4) containing 25 mg % bovine-serum albumin, saturated with a mixture of oxygen and carbon dioxide (95:5) by means a membrane oxygenator with simultaneous temperature adjustment (37 °C). The composition of the Krebs/Henseleit-bicarbonate buffer is the following: 115 mM NaCl, 25 mM NaHCO_3_, 5.8 mM KCl, 1.2 mM Na_2_SO_4_, 1.18 mM MgCl_2_, 1.2 mM NaH_2_PO_4_ and 2.5 mM CaCl_2_. The perfusion fluid enters the liver via the portal vein cannula and leaves the organ through the cava vein cannula. Samples of the effluent perfusion fluid were collected and analyzed for their metabolite contents. Octopamine was added to the perfusion fluid at the desired concentrations (up to 500 μM).

### 3.3. Analytics

In the effluent perfusion fluid the following compounds were assayed by means of standard enzymatic procedures: glucose, lactate and pyruvate [39]. The oxygen concentration in the outflowing perfusate was monitored continuously, employing a Teflon-shielded platinum electrode adequately positioned in a plexiglass chamber at the exit of the perfusate [20]. Metabolic rates were calculated from input-output differences and the total flow rates and were referred to the wet weight of the liver.

The portal perfusion pressure was monitored by means of a pressure transducer (Hugo Sachs Elektronic-Harvard Apparatus GmbH, March-Hugstetten, Germany). The sensor was positioned near the entry of the portal vein, and the transducer was connected to a recorder [40]. The pressure changes were computed from the recorder tracings and expressed as millimeters of mercury (mm Hg).

In those experiments in which [1-^14^C]octanoate or [1-^14^C]oleate were infused for measuring ^14^CO_2_ production, the outflowing perfusate was collected in Erlenmeyer flasks in 2-minute fractions. The Erlenmeyer flasks were rapidly and tightly closed with rubber stoppers to which scintillation vials containing phenylethylamine were fastened by means of stainless steel wires. Trapping of the ^14^CO_2_ in phenylethylamine was accomplished by acidification of the perfusate with a HCl solution which was injected into the flasks through the rubber stoppers. Radioactivity was measured by liquid scintillation spectroscopy. The scintillation solution was: toluene/ethanol (2/1) containing 5 g/L 2,5-diphenyloxazole and 0.15 g/L 2,2-p-phenylene-bis(5-phenyloxazole). The rate of ^14^CO_2_ production was calculated from the specific activity of each labeled fatty acid and from the rate of radioactivity infusion.

### 3.4. Statistics

The error parameters presented in the graphs are standard errors of the means. Statistical analysis was performed with the GraphPad Prism software^®^ (version 6.0; Graph Pad Software, San Diego, CA, USA).

## 4. Conclusions

Octopamine accelerates both carbohydrate and fatty acid metabolism in the rat liver, an action that is in some aspects similar to that reported for the fat body of insects [7]. The action in the rat liver comprises both catabolic (e.g., glycogenolysis, oxidation of fatty acids) and anabolic (e.g., gluconeogenesis) processes via adrenergic stimulation. Acceleration of oxygen uptake under substrate-free perfusion conditions also means acceleration of the oxidation of endogenous fatty acids, which are derived from lipolysis in the liver itself [27]. Stimulation of the oxidation of exogenous fatty acids, on the other hand, is a necessary complement to the increased lipolysis in adipocytes [14] because if fatty acids released from the adipose tissue are not oxidized they will eventually return to the fat depots [41]. Oxidation of fatty acids is expected to occur mainly in the muscles, but our observations suggest that this might also happen in the liver under the influence of octopamine. All these events are compatible with an overall stimulating effect of octopamine on metabolism, which is consistent with its reported weight-loss effects in experimental animals [42]. It is thus likely that octopamine can also contribute for the weight-loss effects of natural products, such as *C. aurantium* extracts [14,15], in which the amine is present. The effects that were found in the present work are also compatible with its supposed stimulating and performance-enhancing properties that lead to its prohibition in sports [34]. The contribution of the liver in this respect resides mainly in enhancing glucose availability to other tissues, muscles for example, via both glycogenolysis and gluconeogenesis. Stimulation of fatty acid oxidation can be equally important as a source of ketone bodies for oxidation in other tissues.
